# Validation of the graded prognostic assessment for lung cancer with brain metastases using molecular markers (lung-molGPA)

**DOI:** 10.1186/s13014-017-0844-6

**Published:** 2017-06-26

**Authors:** Carsten Nieder, Mandy Hintz, Oliver Oehlke, Angelika Bilger, Anca L. Grosu

**Affiliations:** 1 Department of Oncology and Palliative Medicine, Nordland Hospital, 8092 Bodø, Norway; 2 Department of Clinical Medicine, Faculty of Health Sciences, University of Tromsø, 9037 Tromsø, Norway; 3 Department of Radiation Oncology, University Hospital Freiburg, 79106 Freiburg, Germany; 4 German Cancer Consortium (DKTK), Partner Site Freiburg, Freiburg, Germany

**Keywords:** Brain metastases, Lung cancer, Radiotherapy, Prognostic factors

## Abstract

**Background:**

Many patients with brain metastases from non-small cell lung cancer have limited survival, while others survive for several years, depending on patterns of spread, EGFR and ALK alterations, among others. The purpose of this study was to validate a new prognostic model (Lung-molGPA) originally derived from a North American database.

**Patients and methods:**

This retrospective study included 269 German and Norwegian patients treated with individualized approaches, always including brain radiotherapy. Information about age, extracranial spread, number of brain metastases, performance status, histology, EGFR and ALK alterations was collected. The Lung-molGPA score was calculated as described by Sperduto et al.

**Results:**

Median survival was 5.4 months. The score predicted survival in patients with adenocarcinoma histology and those with other types. For example, median survival was 3.0, 6.2, 14.7 and 25.0 months in the 4 different prognostic strata for adenocarcinoma. The corresponding figures were 2.4, 5.5 and 12.5 months in the 3 different prognostic strata for non-adenocarcinoma.

**Conclusions:**

These results confirm the validity of the Lung-molGPA in an independent dataset from a different geographical region. However, median survival was shorter in 6 of 7 prognostic strata. Potential explanations include lead time bias and differences in treatment selection, both brain metastases-directed and systemically.

## Introduction

One of the major challenges in the treatment of non-small cell lung cancer (NSCLC) is the high risk of brain metastases [1]. The continuous improvement of local treatment options, e.g., surgery and radiosurgery, which has paralleled development of better systemic therapies, has resulted in increasingly individualized approaches [2–6]. While some clinicians prefer simultaneous treatment of radiologically visible macroscopic metastases and microscopic disease, others recommend local therapy alone with deferred salvage at the time of progression [7–9]. Efforts are also being made to identify patients who can safely continue systemic therapy without upfront brain radiotherapy, and patients whose prognosis is so poor that best supportive care should be considered [10–14]. Given that brain metastases can occur early or late during the disease trajectory, management decisions are not always simple and straightforward [15].

Prognostic tools have long been used to support decision making and to stratify participants in prospective clinical trials [16–19]. Scores such as the recursive partitioning analysis (RPA) [20] or graded prognostic assessment (GPA) [21, 22] have been validated in several studies and adopted widely [20, 21]. Researchers have realized that these tools should be updated to reflect unique biological features of different primary tumor types, e.g. for breast and lung cancer [23–27]. Specifically for NSCLC a refined score integrating molecular features (EGFR and ALK alterations; Lung-molGPA) has recently been developed by a North American collaborative group, which previously has published the GPA [28]. The purpose of the present study was to validate the Lung-molGPA in an independent European patient population, hypothesizing that a validated score would gain wide acceptance and could replace the older RPA and GPA scores.

## Material and methods

### Patients and treatment

A retrospective study of 269 patients with irradiated brain metastases from NSCLC was performed. Patients managed with best supportive care rather than primary or post-operative radiotherapy were excluded. Treatment was individualized and consisted of focal therapies such as surgery, radiosurgery and stereotactic fractionated radiotherapy with or without whole-brain radiotherapy (WBRT) or upfront WBRT alone with total doses in the range of 20–40 Gy. Patients who failed to complete all fractions of radiotherapy were also included. Salvage treatment of intracranial lesions was individualized, too. All approaches mentioned above were considered at the time of relapse or progression. Systemic treatment was usually prescribed as judged appropriate by the patients’ medical oncologists, both before and after brain-directed treatments. The patients were treated between 2005 and 2015 and identified from a previously described database [19, 29], which includes data from the radiotherapy centers in Bodø and Freiburg. Prognosis was estimated on the basis of age, Karnofsky performance status (KPS), extracranial metastases, number of brain metastases and NSCLC subtype as described in the original publication [28] and shown in Table 1. Differences to the widely used lung cancer-specific GPA score are also shown in the table.

**Table 1:** Baseline characteristics included in the Lung-molGPA (Sperduto et al. 2016 [28]): minimum point sum 0 (poor prognosis), maximum point sum 4 (good prognosis)

Parameter	Lung-molGPA	DS-GPA
Metastatic spread to extracranial sides	0	0
Brain metastases only	1	1
Age ≥70 years	0	0 if >60 years
Age <70 years	0.5	0.5 if 50–60 years, 1 if <50 years
Karnofsky performance status ≤70	0	0 if <70
Karnofsky performance status 80	0.5	0.5 if 70–80
Karnofsky performance status 90–100	1	1
Number of brain metastases >4	0	0 if >3
Number of brain metastases 1–4	0.5	0.5 if 2–3, 1 if 1
EGFR or ALK positive	1	not part of the assessment

*DS-GPA* diagnosis-specific graded prognostic assessment [22]

## Statistical methods

Actuarial survival from the first day of radiotherapy or from surgery was calculated employing the Kaplan-Meier method, and different groups were compared using the log-rank test (SPSS 23, IBM Corp., Armonk, NY, USA). Date of death was known in all patients. A multivariate Cox regression analysis was also performed (forward conditional method) and included all variables with *p*-value ≤0.05 in univariate log-rank tests.

## Results

### Patient characteristics

The median age was 63 years (range 33–85). The median KPS was 80 (range 30–100). The most common initial treatment approach was primary WBRT alone (72%), followed by surgery in combination with post-operative radiotherapy (21%). Further patient characteristics are shown in Table 2.

**Table 2:** Patient characteristics

Parameter	Number	Percent
Male gender	155	58
Female gender	114	42
Adenocarcinoma	192	71
Non-adenocarcinoma	77	29
Extracranial metastases	176	65
No extracranial metastases	93	35
Single brain metastasis	54	20
2–4 brain metastases	86	32
>4 brain metastases	129	48
EGFR or ALK positive	19	7
Age <70 years	200	74
Age ≥70 years	69	26
KPS <70	52	19
KPS 70	70	26
KPS 80	50	19
KPS 90–100	97	36
Upfront whole brain radiotherapy^a^	193	72
Upfront neurosurgery	57	21
Upfront radiosurgery	16	6
Upfront stereotactic fractionated radiotherapy	3	1
Supportive care alone	0	0

*KPS* Karnofsky performance status

^a^includes patients with delayed (salvage) neurosurgery, radiosurgery, fractionated re-irradiation

### Lung-molGPA

**Fig. 1:**
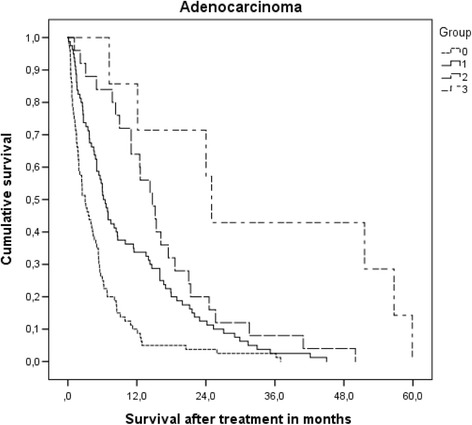
Actuarial survival of patients with adenocarcinoma stratified by Lung-molGPA, *p* = 0.0001 (pooled over all strata)

**Fig. 2:**
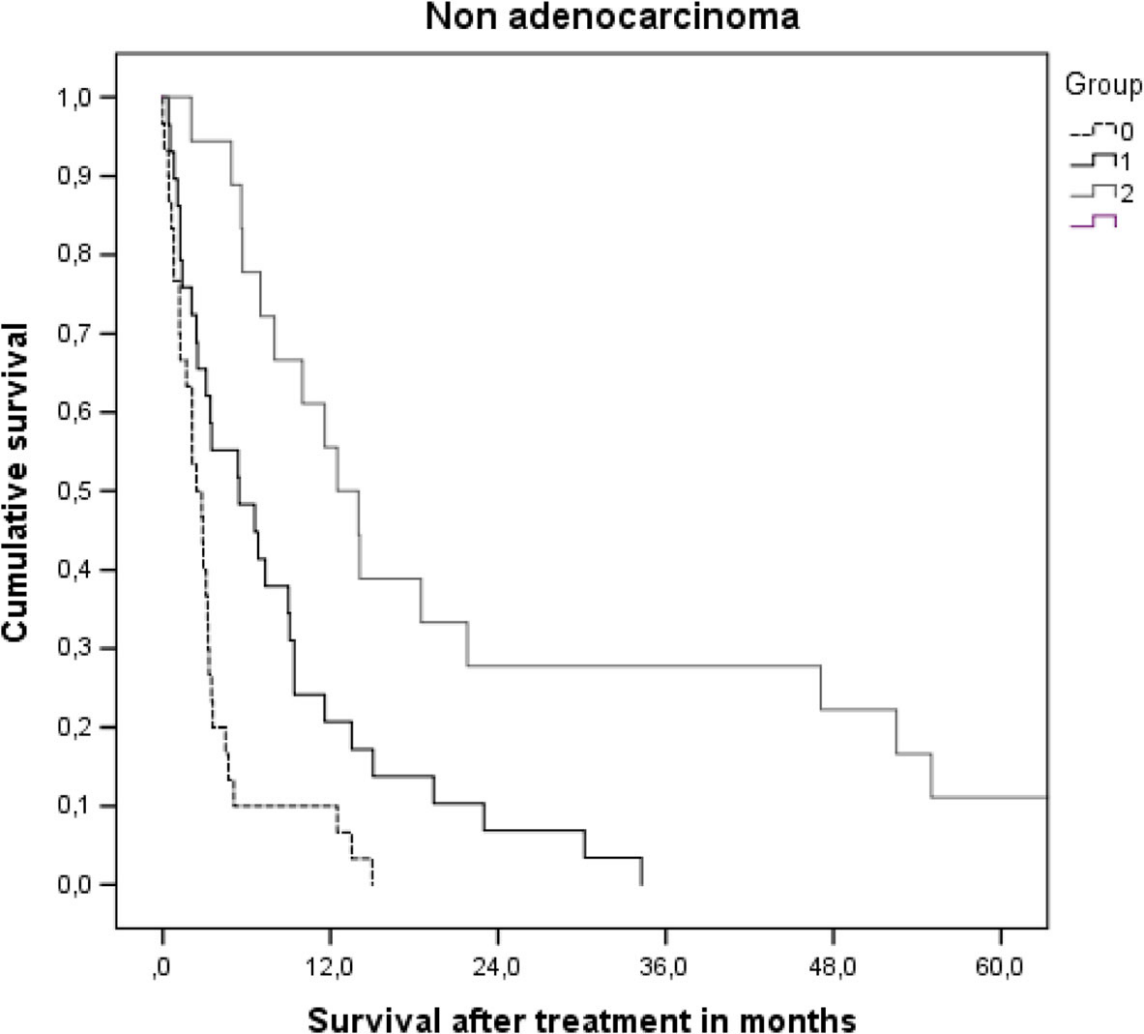
Actuarial survival of patients with non-adenocarcinoma stratified by Lung-molGPA, *p* = 0.0001 (pooled over all strata)

Most patients had unfavorable prognostic features, i.e. 0–1 point in 110 patients (41%) and 1.5–2 points in 109 (41%). Forty-two patients (16%) had 2.5–3 points and the remaining 8 (3%) had 3.5–4 points. These four prognostic strata had significantly different median survival of 2.8, 6.2, 14.0 and 25.0 months (*p* < 0.0001, log-rank test pooled over all strata). Overall median survival was 5.4 months. Table 3 shows the results of univariate prognostic factors for survival. In multivariate Cox regression analysis KPS (dichotomized variable as in [28], *p* = 0.0001), extracranial metastases (*p* = 0.002), age (dichotomized variable as in [28], *p* = 0.05), EGFR or ALK alteration (*p* = 0.001) and number of brain metastases (dichotomized variable as in [28], *p* = 0.05) were significant predictors of survival. Figure 1 and Table 4 show the survival outcomes of patients with adenocarcinoma. Figure 2 and Table 4 show the corresponding data in case of non-adenocarcinoma histology.

**Table 3:** Univariate analysis of prognostic factors for overall survival (log-rank test)

Parameter	Median survival in months	*p*-value
Male gender	5.5	
Female gender	5.0	0.49
Adenocarcinoma	5.6	
Non-adenocarcinoma	4.5	0.87
Extracranial metastases	4.4	
No extracranial metastases	7.5	0.0001
1–4 brain metastases	6.0	
>4 brain metastases	4.6	0.04
EGFR or ALK positive	22.9	
Not EGFR or ALK positive	5.0	0.0001
Age <70 years	6.0	
Age ≥70 years	3.0	0.006
KPS ≤70	2.5	
KPS 80	7.0	
KPS 90–100	11.0	0.0001

*KPS* Karnofsky performance status

**Table 4:** Survival outcomes stratified by Lung-molGPA

Group	Number	Median survival in months	6-month probability	12-month probability
Adeno 0–1 p.	80	3.0	26	10
Adeno 1.5–2 p.	80	6.2	54	34
Adeno 2.5–3 p.	25	14.7	84	64
Adeno 3.5–4 p.	8	25.0	100	88
Other 0–1 p.	30	2.4	10	10
Other 1.5–2 p.	29	5.5	48	21
Other 2.5–3p.	18	12.5	78	56

## Discussion

We performed a retrospective validation study of the Lung-molGPA [28] in a European patient population, comparable to the previous validation of the DS-GPA [29]. The study population consisted mainly of patients with intermediate or poor prognosis who were judged not to be appropriate candidates for aggressive local therapies, such as surgery or stereotactic radiotherapy, but received active brain-metastases-directed therapy. This discrepancy likely explains why the median survival in our study was 5.4 months, while the North American patients survived for a median of 15.2 months (adenocarcinoma) and 9.2 months (non-adenocarcinoma). Other treatments (chemotherapy, targeted drugs, salvage of brain metastases) might have differed too, however, they were not recorded in any of the studies. Neither time interval from initial cancer diagnosis to brain metastases nor diagnostic setting (imaging in asymptomatic patients vs. clinical deficits) has been evaluated, resulting in potential lead time bias if North American patients were treated earlier. In principle, imbalances of patient characteristics such as KPS or mutation status could have contributed to the survival difference. However, median survival was shorter in 6 of 7 prognostic strata in our study. For example, patients with adenocarcinoma had inferior survival in all 4 strata (median 3.0 vs. 6.9 months; median 6.2 vs. 13.7 months; median 14.7 vs. 26.5 months; median 25.0 vs. 46.8 months). For non-adenocarcinoma the following differences emerged: median 2.4 vs. 5.3 months, median 5.5 vs. 9.8 months, and median 12.5 vs. 12.8 months. Most of these differences are clinically relevant and we therefore recommend additional studies in patients managed with different approaches in different regions of the world. The main result of our study was that the Lung-molGPA accurately reflects the prognostic impact of different baseline characteristics. This score seems to represent a useful improvement of its widely adopted ancestors such as RPA and DS-GPA [20–22].

Limitations of this study, which followed the methods used by Sperduto et al. [28], include the number of patients, statistical power of subgroup analyses, and retrospective design. Whereas the number of patients was limited in our database, they represent the total cohort of the two radiotherapy departments and consequently express the daily practice at these academic hospitals. Given that patients managed with best supportive care were excluded, worse survival outcomes could be expected if one would analyze all patients with a brain metastases diagnosis, or in radiation oncology practices that would have offered WBRT to patients who were managed with best supportive care at the two institutions that participated in this study. The North American database included 2186 patients treated between 2006 and 2014. Radiosurgery was a component of care in more than 50% of patients with adenocarcinoma. In addition, neurosurgical resection was performed in selected patients. In our study, less than 30% received upfront surgery, radiosurgery or stereotactic fractionated radiotherapy. We have previously reported that increasing use of focal treatments such as radiosurgery and surgical resection and also of systemic treatment has resulted in prolonged survival, especially for patients with favorable prognostic features [30]. The observed survival differences between the present study and the one reported by Sperduto et al. [28] are in line with the hypothesis that continuous improvements of multimodal care translate into better outcome. With the advent of targeted drugs with high efficacy in molecularly-defined subgroups [10, 11, 31], and possibly also immunotherapy [32], further improvement can be expected.

## Conclusions

The data presented in this study confirm the validity of the Lung-molGPA in patients from a different geographical region. However, median survival was shorter in 6 of 7 prognostic strata. Potential explanations include differences in treatment selection, both brain metastases-directed and with systemic agents. These hypotheses require additional studies.

## Abbreviations

ALK: Anaplastic lymphoma kinase
EGFR: Epidermal growth factor receptor
GPA: Graded prognostic assessment
KPS: Karnofsky performance status
NSCLC: Non-small cell lung cancer
RPA: Recursive partitioning analysis
WBRT: Whole brain radiotherapy
